# Progressive Neuronal Degeneration Leading to Impaired Motor Function: A Case of Alcohol-Induced Peripheral Neuropathy

**DOI:** 10.7759/cureus.93207

**Published:** 2025-09-25

**Authors:** Aida R Mouzoon, David H Rustom

**Affiliations:** 1 Physical Medicine and Rehabilitation, Wayne State University School of Medicine, Detroit, USA; 2 Pain Management, Wayne State University Detroit Medical Center, Detroit, USA

**Keywords:** acquired peripheral neuropathy, chronic alcoholic, emg-ncv, neuropathic pain treatment, peripheral neuropathy management

## Abstract

Alcoholic peripheral neuropathy involves degeneration of the peripheral motor neurons, typically in the lower extremities, accompanied by painful sensations and impairments in gait as a direct result of alcohol’s toxicity on the nervous system. Symptoms can range from mild to severe, and many factors, such as use history, comorbidities, lifestyle, and family history, determine the disease course and success of treatment. Laboratory values include deficiencies in many key nutrients for neuron health, including vitamin B1 (thiamine), vitamin B12, and folic acid. Here, we discuss a case of a 31-year-old female patient who presented with symptoms of severe peripheral neuropathy, including paresthesias, pain, burning, and gait impairment, as a result of years of alcohol use. Following a diagnosis of peripheral neuropathy, complete alcohol cessation and symptom management are indicated as treatment. The patient’s regimen includes a multifactorial, supportive approach with neuropathic, opioid, and anti-inflammatory medications in addition to physical therapy and lifestyle changes to keep a stable baseline.

## Introduction

Alcohol-induced peripheral neuropathy (ALN), also known as alcoholic neuropathy, is a progressive disease of neuronal damage leading to lasting and sometimes permanent deficits in the function of the peripheral nervous system. This is a common adverse effect of chronic alcohol consumption, and patients typically present with pain, ataxia, and paresthesia in the lower extremities [1]. Symptom manifestation is gradual and occurs due to malnutrition and the resulting vitamin deficiencies thereof. Most notably, vitamin B1 (thiamine), a key nutrient inhibited by chronic alcohol consumption, serves as a coenzyme in neuron development [1], and a lack of vitamin B1 results in poor neuron health and subsequently damage. Neurons are permanent tissues, meaning they do not have an infinite capacity to regenerate like other cells in our bodies do. As a result, many patients struggle with symptom management and finding relief with the therapies currently available. The clinical workup for alcoholic neuropathy involves a combination of medical and family history, physical exam findings, nerve function testing, and blood work. Patients will see deficits in the most distal parts of the body, i.e., the lower extremities, given that axonal propagation is impaired. Common exam findings include sensory, motor, autonomic, and gait abnormalities, followed by painful sensations that may present with a burning quality, and sensations of weakness [2]. Following symptom presentation, patients will typically undergo an electromyogram (EMG), which is a nerve function test to assess the electrical activity of nerves, in both upper and lower extremities. This test can pinpoint the exact dermatomes and nerve roots that can be impaired in this disease process. Typical EMG findings in alcoholic neuropathy patients include reduced recruitment pattern of motor units and active denervation, more so affecting the lower extremities [3]. Given the spectrum of severity for this disease, therapy for these patients requires a multifactorial symptom-based approach with opioids, neuropathic pain medications, non-steroidal anti-inflammatory drugs (NSAIDs), B1 supplementation, and physical therapy. We will present a case of severe ALN as a result of years of chronic alcohol abuse in conjunction with uncontrolled diabetes and a positive family history of alcohol abuse. Even with a multimodal approach, lifestyle changes, and treatment compliance, a successful therapeutic response can be difficult to achieve, given the severity of her disease.

## Case presentation

A 31-year-old woman was evaluated in the clinic for many years of gradual, progressive peripheral neuropathy. She had a several-year-long history of daily alcohol use and has been sober for one year. The patient had a history of previous uncontrolled diabetes and endorsed a positive family history of alcohol abuse. She reported symptoms of paresthesia, burning, numbness, and tingling (in her extremities) that interfered with her daily activities. She described the pain as sharp and stabbing in nature, 7/10 on average, and 10/10 with strenuous exertion and standing. During the past six weeks, she engaged in physical therapy, to which she endorsed only mild alleviation in her pain, in addition to medical therapy with gabapentin 600 mg three times a day (TID), NSAIDs, and duloxetine 30 mg daily. Concurrently, she adjusted her lifestyle habits and compliance in terms of controlling her diabetes with semaglutide and insulin, with blood sugar ranging from 110 to 170 mg/dL (reference: <180 mg/dL for diabetics). She denied illicit substance use but endorsed daily nicotine use with a vape. Physical examination revealed reduced sensation and proprioception in the bilateral lower extremities in a stocking-like distribution, with zero proprioception of the great toes bilaterally. There was no intrinsic muscle loss on physical exam. The patient’s diagnosis of alcoholic neuropathy was confirmed with her prior history, loss of lower extremity proprioception, and bilateral peripheral paresthesias. Her condition slightly improved following lifestyle modifications and neuropathic pain medications. Regarding cognitive and behavioral adjustments, her progress will be supported by a neuropsychologist to aid with long-term strategies for sobriety adherence. Refractory treatment for this patient includes stimulation with a spinal cord or peripheral nerve stimulator.

## Discussion

Alcohol’s toxicity manifests from the micro to macro level, targeting many biochemical pathways that contribute to nerve health and overall motor function. Alcohol, or ethanol metabolism, is a three-step oxidative pathway involving two enzymes and one cofactor: alcohol dehydrogenase, aldehyde dehydrogenase, and nicotinamide adenine dinucleotide (NAD), respectively. The cofactor NAD is crucial in many metabolic processes, serving as a shuttle for electrons and protons, thus facilitating many reactions that are important for homeostasis. Alcohol’s metabolism is contingent on the availability of NAD+ to be reduced to nicotinamide adenine dinucleotide hydrogen (NADH), and in a chronic state of consumption, this can be limited, thus halting alcohol oxidation. The main reservoir of NAD+ stems from mitochondrial respiration, also known as the electron transport chain, and when this process cannot keep up with recycling cofactors, mitochondrial injury ensues [4]. The mitochondria are impermeable to NADH; therefore, they rely on different reducing equivalents to later be converted to NAD+ to keep forward flow going to generate energy and cofactors [4]. NAD+ levels naturally decline as a result of normal aging; however, when there is an external irritant, increasing the demand as alcohol does, the body begins to adapt to a proinflammatory state.

**Figure 1:**
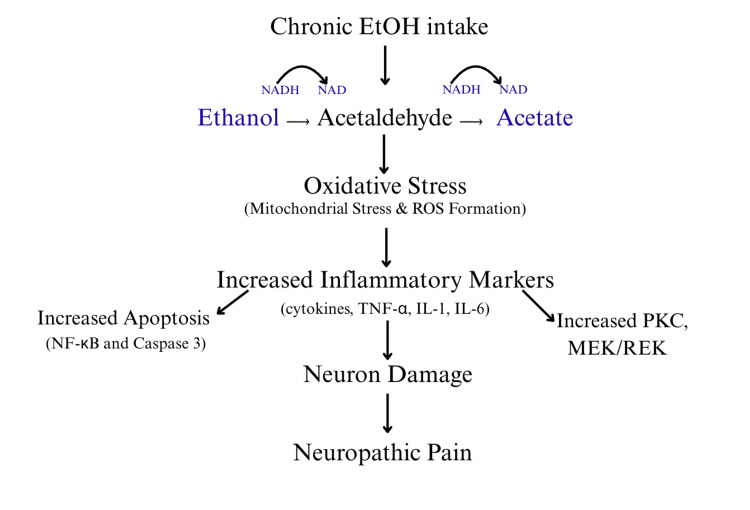
The intracellular effects of chronic alcohol consumption and its role in neuropathic manifestations. The biochemical cascade triggered by chronic alcohol use and its activation of proinflammatory signaling markers that leads to neuronal degeneration. EtOH: alcohol; NAD/NADH: nicotinamide adenine dinucleotide (+ hydrogen); ROS: reactive oxygen species; TNF-a: tumor necrosis factor alpha; IL: interleukin; NF-kB: nuclear factor kappa-light-chain-enhancer of activated B cells; PKC: protein kinase C. Image Credit: Aida Mouzoon

This new steady-state triggers the immune system, activating inflammatory cascades as a way to compensate for the body’s inability to flush out this toxin. Chronic inflammation activates communicating proteins, or cytokines, which mediate various inflammatory responses by signaling to other immune markers. One important system, called the toll-like receptor system, signals the brain to release microbial products in response to toxins, which stimulates cytokine release in the form of immunoglobulins, tumor necrosis factor-α (TNF-α), and interleukins (IL-1 and IL-6) [5]. These pro-inflammatory mediators circulate in the body as an immune response, and therefore, the mechanism of chronic disease is intrinsically linked with chronic inflammation. This results in systemic organ manifestations; however, in terms of nerve damage, it is the chronic inflammatory state in conjunction with chronic nutrient deficiency that results in motor impairments. Alcohol is very caloric, and its metabolism often results in malnutrition, as it inhibits the absorption of many vitamins, including B1, B6, B12, folic acid, and zinc. Most notably, the B vitamins play a crucial role in overall nerve health with B1 as an antioxidant, B6 in nerve metabolism, and B12 in protecting the myelin sheath, and as a result are called “neurotropic” vitamins [6]. Chronic disease states, such as alcoholism, suppress nerves from receiving nutrients, thus affecting nerve health, leading to degeneration. Nerve fibers have the capacity to regenerate to a certain extent, recruiting immune macrophages to carry away myelin and dead cells, in the presence of B vitamins that support and enable this process [6]. When the regenerative capacity of nerves depletes, there is minimal room for regeneration, as the nerves are permanent tissues and do not carry the stem cell capacity to repair like other tissues (Figure 1).

At this point, nerve damage ensues as a result of irreversible apoptosis or cell death, leading to neuropathic symptoms within a patient. The onset of symptoms is described as gradual and progressive. Patients with ALN typically present with sensory, motor, and autonomic dysfunctions, including hyperalgesia, paresthesia, pain in the extremities, gait abnormalities, and weakness [2,7]. Cognitive symptoms, such as confusion and memory impairments, and in extreme cases, Korsakoff’s or Wernicke's encephalopathy, may also present alongside peripheral symptoms. Electromyogram and nerve conduction studies can help conclude a diagnosis of ALN, with studies showing denervation, slowed conduction velocity up to 60% of normal, in addition to loss of small and large fibers, reduction in amplitude of sensory potentials, and overall axonal loss of myelinated and unmyelinated fibers [8]. The combination of these findings explains the motor and sensory deficits that arise in these patients. MRI can be used as an additional imaging modality to assess the progression of degenerative changes in the brain and extremities. In a study of 31 patients with excessive alcohol use, microstructural markers, including sensitive proton spin density (⍴) and more specific T2 relaxation time (T2app), were used to assess changes in peripheral nerve tissues [9]. These markers measure the mobility of protons and how quickly the synchronicity of their spin is lost, also known as phase coherence [9]. Researchers found that ALN patients had increased ⍴ and prolonged T2app, suggesting increased water content as a result of chronic inflammation and demyelination [9]. Additional factors such as cross-sectional area and T2-weighted signal (T2ws) were also assessed; however, there was no significance between ALN patients and healthy controls [9]. In the central nervous system, axons and myelin compose the white matter of the brain, allowing nerve signals to rapidly transmit within various brain regions, and often across hemispheres. However, in ALN, this is impaired, leading to the many cognitive manifestations of this disease. MRIs of patients with ALN confirm these changes with a reduction in white matter volume within the brain, in addition to gray matter damage [10]. MRI is useful as an additional imaging modality; however, nerve conduction studies, clinical history, and physical exam are the frontline for diagnosing ALN.

Complete alcohol cessation and supplementation of B vitamins are crucial in the preliminary treatment of ALN. One study found that supplementation with benfotiamine, a synthetic thiamine derivative, increases levels of active-form thiamine diphosphate in animal models, leading to a potential increase in sensory and motor perception [2]. Standard of care for ALN involves a multimodal approach, with medication and lifestyle modifications. For symptoms of paresthesias, numbness, and tingling, gabapentinoids are used off-label for neuralgia, including gabapentin and pregabalin. Alternatively, the use of amitriptyline and duloxetine is also indicated for neuropathic pain control. Side effects of these medications include brain fog, and often patients need to be uptitrated for neuropathic properties to take effect. In conjunction with neuropathic medications, many patients are on pain medications in the form of NSAIDs or opioids such as hydrocodone-acetaminophen (Norco) or oxycodone-acetaminophen (Percocet). Contraindications to these medications include tolerance and substance use history, especially in patients with an alcohol use history; therefore, another alternative includes partial agonism with buprenorphine sublingual films or as a transdermal patch. Many patients additionally benefit from physical therapy and lifestyle changes to keep baseline strength in their extremities. However, patients with prolonged disease and delay in accessing care may need a refractory solution with central or peripheral nerve stimulation. Neuromodulation can be in the form of a spinal cord stimulator or a peripheral nerve stimulator, in which the former administers electrical impulses to block pain signals, and the latter targets smaller, specific nerve plexuses outside the spinal cord. Both options involve minimally invasive surgical implantation and coordination with device experts to find optimal settings for long-term pain control. Pain management with an intrathecal pain pump or Prialt can also serve as a more precise alternative, as it delivers microdoses of various pain medications via a catheter that acts directly on the nerve fiber. Different medication concoctions are often trialed, and the regimen varies from patient to patient. Overall, these therapies serve as an alternative to oral medications, but they entail their own set of limitations and can be cost-prohibitive; hence, they are refractory.

## Conclusions

Alcohol-induced peripheral neuropathy is a progressive and degenerative disease of motor neurons that can lead to significant and often irreversible impairments in function and quality of life. Many factors, including alcohol use history, lifestyle, comorbidities, and genetics, play a role in the severity of this condition as well as response to treatment. The disease results in nerve damage manifesting as numbness, tingling, pain, and gait abnormalities in the extremities, most notably the lower extremities. In addition to a positive history of alcohol abuse, a thorough physical examination and workup, including nerve function testing and blood work, will confirm the diagnosis of peripheral neuropathy. Following diagnosis, patients respond differently to treatment due to the aforementioned factors; however, typical regimens to keep patients functional and stable include neuropathic, opioid, and NSAID pain management, physical therapy, and complete alcohol cessation.
